# Estimation of 24-Hour Urinary Sodium Excretion Using Spot Urine Samples

**DOI:** 10.3390/nu6062360

**Published:** 2014-06-20

**Authors:** Moo-Yong Rhee, Ji-Hyun Kim, Sung-Joon Shin, Namyi Gu, Deuk-Young Nah, Kyung-Soon Hong, Eun-Joo Cho, Ki-Chul Sung

**Affiliations:** 1 Cardiovascular Center, Dongguk University Ilsan Hospital, 27 Dongguk-ro, Ilsandong-gu, Goyang-si, Gyeonggi 410-773, Korea; E-Mail: mdangela79@gmail.com; 2 Clinical Trial Center, Dongguk University Ilsan Hospital, 27 Dongguk-ro, Ilsandong-gu, Goyang-si, Gyeonggi 410-773, Korea; E-Mail: namyi.gu@gmail.com; 3 Division of Nephrology, Dongguk University Ilsan Hospital, 27 Dongguk-ro, Ilsandong-gu, Goyang-si, Gyeonggi 410-773, Korea; E-Mail: shine@dumc.or.kr; 4 Department of Clinical Pharmacology and Therapeutics, Dongguk University Ilsan Hospital, 27 Dongguk-ro, Ilsandong-gu, Goyang-si, Gyeonggi 410-773, Korea; 5 Cardiovascular Center, Dongguk University Gyeongju Hospital, 87 Dongdae-ro, Seokjang-dong, Gyeongju-si, Gyeongbuk 780-350, Korea; E-Mail: ptca@dongguk.ac.kr; 6 Department of Cardiology, Hallym University College of Medicine, 77 Sakju-ro, Chuncheon-si, Gangwon-do 200-704, Korea; E-Mail: kshong@hallym.ac.kr; 7 Department of Cardiology, The Catholic University of Korea College of Medicine, 222 Banpodae-ro, Seocho-gu, Seoul 137-701, Korea; E-Mail: choej4oct@gmail.com; 8 Department of Internal Medicine, Kangbuk Samsung Hospital, Sungkyunkwan University School of Medicine, 29 Saemunan-ro, Jongro-Ku, Seoul 110-746, Korea; E-Mail: kcmd.sung@samsung.com

**Keywords:** sodium intake, spot urine, 24-hour collection

## Abstract

The present study evaluated the reliability of equations using spot urine (SU) samples in the estimation of 24-hour urine sodium excretion (24-HUNa). Equations estimating 24-HUNa from SU samples were derived from first-morning SU of 101 participants (52.4 ± 11.1 years, range 24–70 years). Equations developed by us and other investigators were validated with SU samples from a separate group of participants (*n =* 224, 51.0 ± 10.9 years, range 24–70 years). Linear, quadratic, and cubic equations were derived from first-morning SU samples because these samples had a sodium/creatinine ratio having the highest correlation coefficient for 24-HUNa/creatinine ratio (*r =* 0.728, *p <* 0.001). In the validation group, the estimated 24-HUNa showed significant correlations with measured 24-HUNa values. The estimated 24-HUNa by the linear, quadratic, and cubic equations developed from our study were not significantly different from measured 24-HUNa, while estimated 24-HUNa by previously developed equations were significantly different from measured 24-HUNa values. The limits of agreement between measured and estimated 24-HUNa by six equations exceeded 100 mmol/24-hour in the Bland-Altman analysis. All equations showed a tendency of under- or over-estimation of 24-HUNa, depending on the level of measured 24-HUNa. Estimation of 24-HUNa from single SU by equations as tested in the present study was found to be inadequate for the estimation of an individual’s 24-HUNa.

## 1. Introduction

Sodium is an essential nutrient to maintain physiological homeostasis. However, excessive sodium intake is associated with elevation of blood pressure and greater risk of cardiovascular disease and stroke [1,2]. Thus, reduction of sodium intake is a well-established public health issue. 

In the evaluation and management of disease affected by net sodium balance, accurate assessment of sodium intake is crucial. In general, this is accomplished by dietary survey and 24-hour urine collection method. Although measurement of 24-hour urine sodium excretion (24-HUNa) is considered the most reliable method [3], the high burden of labor and difficulty in complete collection has prompted the search for more practical methods, such as a spot urine (SU) sample analysis and overnight urine collection. Several methods of estimating 24-hour urine sodium excretion from SU samples have been developed and used as an alternative to a 24-hour urine collection method [4,5,6,7]. However, estimation of 24-HUNa using SU samples is questionable and its reliability has been poorly validated in the assessment of individual sodium intake.

In the present study, we developed and validated equations using SU samples to estimate 24-HUNa. In addition, we also validated previously proposed equations.

## 2. Experimental Section

### 2.1. Study Subjects and Urine Sampling Protocols

For the purpose of sodium intake estimation in a general Korean population, participants were recruited by list-assisted random-digit dialing (LARDD) [8,9] in four cities. SU and 24-hour urine samples were collected. Pregnant women, women currently menstruating, individuals with severe liver or kidney disease, and others unable to collect 24-hour urine, were all excluded. Study participants visited study-performing clinical trial centers in respective cities before 08:30 h to receive detailed instruction regarding the study procedure. Fasting blood glucose, total cholesterol, triglyceride, high-density lipoprotein cholesterol, low density lipoprotein cholesterol, serum sodium and potassium levels were measured after at least 8 h of overnight fasting. They were given a 3 L urine collection bag, a 600 mL plastic cup, and three 50 mL Falcon tubes. The tubes were used to collect SU samples.

Urine collection was started after voiding and discarding the first urine sample between 09:00 and 09:30 h. Subsequently, all urine voided during the next 24 h was collected. Participants were asked to maintain their usual life routine. The next day, the participants visited the clinical trial centers before 09:00 h. The last urine voided 24-hour after the first voiding was collected and added to the urine collection bag. During the 24-hour urine collection, three SU samples were obtained. The evening SU sample was collected between 2100–2300 h. The first-morning SU sample was collected from first voided morning urine of the second day of urine collection. The morning random SU sample was collected subsequent to the early morning void around 0900 h. Participants were educated to void in the plastic cup provided. About 10 mL of voided urine in the plastic cup was transferred to a Falcon tube, and the rest of the urine sample in the cup was added to the urine collection bag.

The collected urine samples from the four centers were transferred to the central laboratory (Green Cross LabCell, Yongin, Korea). Sodium concentrations were measured by an ion-selective electrode method (Modular DPE chemistry; Roche Diagnostics, Mannheim, Germany). Creatinine levels were measured by the Jaffe reaction (Kinetic colorimetric assay; Roche Diagnostics). For the calculation of 24-hour urine sodium excretion, the amount of spot urine sample was also added to the total volume for 24-hour.

The validity of 24-hour urine collection was assessed by a combination of self-reported urine loss and the creatinine-based 24-hour urine determination. If a self-reported loss of urine sample was more than 100 mL or more than once, or if a creatinine index [24-hour urine creatinine, mg/dL/(21 × body weight)] was lesser than 0.7 [9,10], the collected urine sample was considered insufficient for a 24-hour period collection.

Hypertension was defined as a 24-hour average systolic blood pressure (SBP) ≥ 130 mmHg or a 24-hour average diastolic blood pressure (DBP) ≥ 80 mmHg, [11] or a current use of antihypertensive medications with a previous diagnosis of hypertension.

Of the 502 participants, urine samples from 367 participants were determined as valid 24-hour urine collection. Three spot urine samples were collected from 101 participants (group 1), and used in the development of equations to estimate 24-HUNa. Among the remainders, 224 participants (group 2) had collected first-morning SU samples, and these samples were used to test the validity of the equations developed in the present study and previously by other investigators. Forty-two participants were excluded because spot urine samples were unavailable.

### 2.2. Development and Validation of Equations

The equation for the estimation of 24-HUNa from SU samples was developed using a process similar to those in previous studies based upon the same hypothesis [4,5].

(1) 24-hour urine creatinine excretion (24HUCr) ≈ predicted 24-hour urine creatinine excretion (PrUCr)
(2) 24-HUNa/24HUCr ∞ spot urine sodium (SUNa)/spot urine creatinine (SUCr)
(3) 24-HUNa ∞ SUNa/SUCr × PrUCr

Since 24HUCr is affected by age, body weight and height [12], the equation for PrUCr from age, body weight and height was developed by a regression analysis between 24HUCr and SUCr from the group 1 subjects. The equations for PrUCr developed by us and other investigators [4,5,13], was validated with the urine sample data provided by group 2 participants. Among the three equations for PrUCr, the equation proposed by Kawasaki *et al*. [4,13], which has gender specific equations, showed the highest correlation coefficient to 24HUCr (*r =* 0.812, *p <* 0.001). The correlation coefficient of equations for PrUCr developed by us (−143.349 − (2.548 × age) + (24.475 × body weight) − (0.320 × height)) was 0.764 (*p <* 0.001), and by Tanaka *et al*. [5] was 0.708 (*p <* 0.001). Thus, in the development of our equations estimating 24-HUNa from SU samples of group 1, we used the equation for PrUCr proposed by Kawasaki *et al*. [4,13], which reflected the difference of gender in urine creatinine excretion [14], instead of using the presently developed equation for PrUCr. 

With the hypothesis that the 24-HUNa is proportional to the ratio of sodium to creatinine in SU multiplied by PrUCr [4], XNa was calculated as: XNa = (SUNa/SUCr) × PrUCr, where PrUCr for men is (body weight × 15.1 + height × 7.4 − age × 12.4 − 80) and for women is (body weight × 8.6 + height × 5.1 − age × 4.7 − 75) [4,13]. The equation estimating 24-HUNa from SU was developed by curve fitting between XNa of first-morning SU, and 24-HUNa. Linear, quadratic and cubic equations were selected. The equations developed presently and previously [4,5,7] were validated with the first-morning voided SU samples of group 2 participants. 

### 2.3. Statistical Analyses

The correlations of SUNa/SUCr against 24-HUNa/24HUCr, and estimated 24-HUNa against measured 24-HUNa were assessed by the Pearson correlation coefficient (r). The differences between measured and estimated 24-HUNa were tested by paired *t*-test. The Bland-Altman analysis of agreement was used to estimate the bias and limits of agreement between measurements by two methods. The percent error was calculated as 1.96 × (standard deviation of the difference between measured and estimated 24-HUNa)/mean of measured 24-HUNa × 100. Statistical analyses were performed using SPSS version 20 (IBM, Chicago, IL, USA) and SigmaPlot version 12.0 (Systat Software, LaJolla, CA, USA). Curve fitting was performed using TableCurve 2D version 5.01 (Systat Software, LaJolla, CA, USA). Null hypotheses of no difference were rejected if the respective *p* value was <0.05.

## 3. Results

### 3.1. Development of Equations Estimating 24-HUNa

The baseline demographic and clinical characteristics of subjects are shown in Table 1. Among group 1 subjects, 43 (42.6%) had hypertension and 17 were receiving antihypertensive drugs. 

**Table 1:** Demographic and clinical characteristics of the study population.

Variables	Group 1	Group 2
	*N =* 101	*N =* 224
Age (years)	52.4 ± 11.1	51.0 ± 10.9
Men (%)	51 (50.5)	89 (39.7)
Hypertensives (%)	43 (42.6)	107 (47.8)
Antihypertensives (%)	17 (16.8)	47 (21.0)
Diabetes (%)	8 (7.9)	11 (4.9)
Body weight (kg)	63.9 ± 10.0	63.5 ± 11.4
Height (cm)	163.4 ± 8.4	162.7 ± 7.6
Serum sodium (mmol/dL)	140.2 ± 2.7	140.4 ± 2.4
Serum potassium (mmol/dL)	4.3 ± 0.4	4.3 ± 0.4
Serum creatinine (mg/dL)	0.80 ± 0.15	0.80 ± 0.16
24-hour urine sodium (mmol/24-hour)	151.4 ± 61.6	165.3 ± 65.5

Data are expressed as mean ± standard deviation, or numbers and percentages in parentheses, as appropriate.

**Figure 1:**
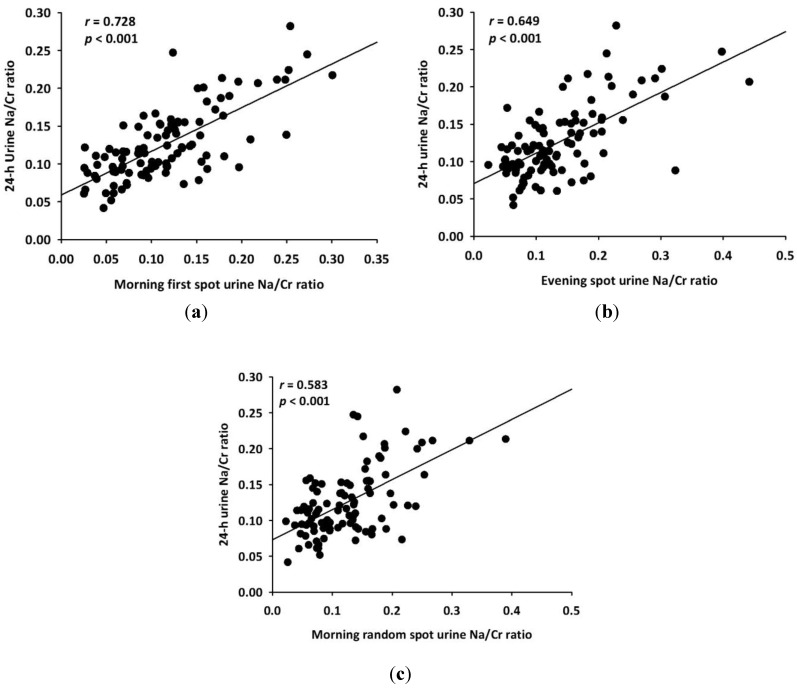
Relationship between (**a**) 24-hour urine sodium/creatinine (Na/Cr) ratio *vs*. first-morning spot urine Na/Cr ratio; (**b**) 24-hour urine Na/Cr ration *vs*. evening spot urine Na/Cr ratio; and (**c**) 24-hour urine Na/Cr ration *vs*. random morning spot urine Na/Cr ratio in 101 subjects.

Among the three spot urines, first-morning SUNa/SUCr showed the highest correlation coefficient to 24-HUNa/24HUCr (*r =* 0.728, *p <* 0.001). The correlation coefficient of evening and morning random SUNa/SUCr were 0.649 (*p <* 0.001) and 0.583 (*p <* 0.001), respectively (Figure 1). 

**Figure 2:**
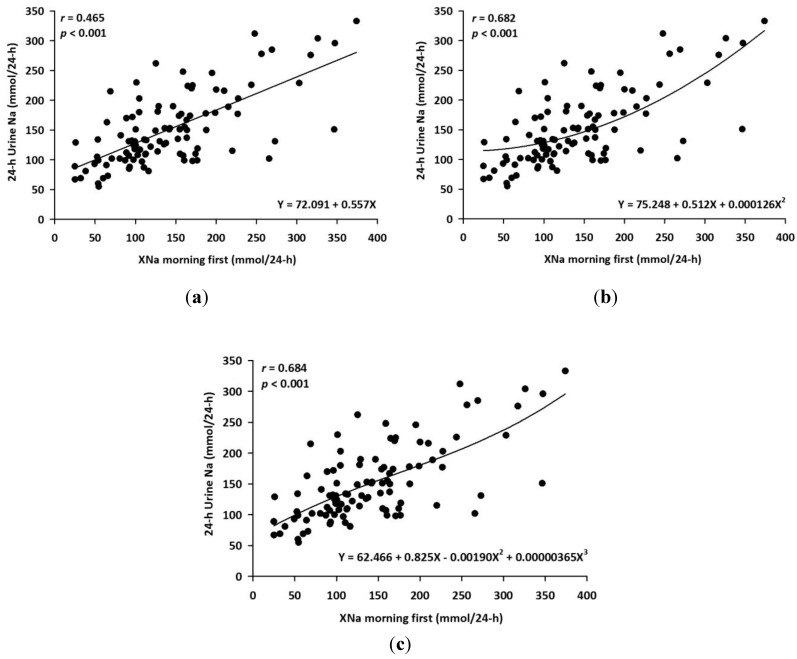
Development of equations by curve fitting from 101 subjects. (**a**) Linear equation association; (**b**) quadratic equation association and (**c**) cubic equation association. Each equations and their correlation coefficient are showed. Equation XNa = (spot urine sodium/spot urine creatinine) × predicted 24-hour urine creatinine excretion.

Thus, first-morning SU was used in the development of the equation for the estimation of 24-HUNa. The best fitting curves of linear, quadratic and cubic equations were obtained as follows (Figure 2):

[Linear equation: estimated 24-HUNa = 72.091 + 0.557 × XNa]
(1)

[Quadratic equation: estimated 24-HUNa = 75.248 + 0.512 × XNa + 0.000126 × XNa^2^] (2)

[Cubic equation: estimated 24-HUNa = 62.466 + 0.825 × XNa − 0.00190 × XNa^2^ + 0.00000365 × XNa^3^] (3)

### 3.2. Validation of Equations Estimating 24-HUNa

First-morning SU samples from group 2 participants were used in the validation of the equations derived presently and previously [4,5,7]. Among the group 2 participants, 107 (47.8%) had hypertension and of these, 47 were receiving antihypertensive drugs.

**Figure 3:**
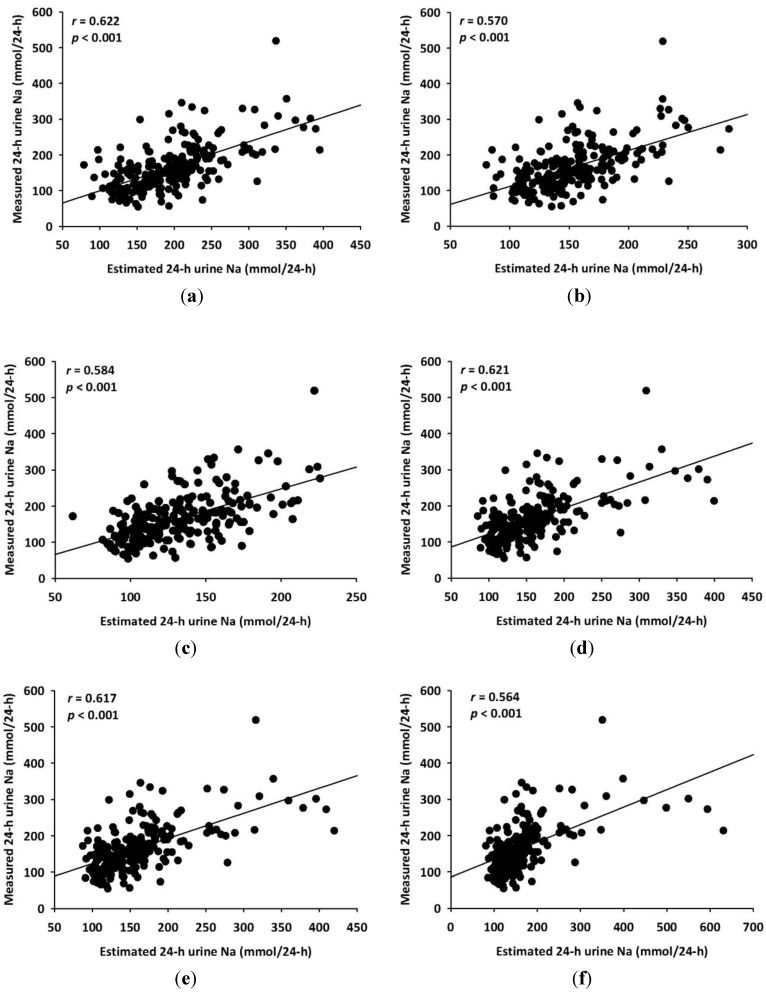
Relationship between measured and estimated 24-hour urine sodium obtained by six equations. (**a**) Kawasaki’s equation; (**b**) Tanaka’s equation; (**c**) INTERSALT equation; (**d**) linear equation; (**e**) quadratic equation; and (**f**) cubic equation.

All of the estimated 24-HUNa using the six equations (three equations from this study and three equations from the aforementioned studies) revealed statistically significant correlations with measured 24-HUNa (Figure 3). 

The estimated 24-HUNa from our 3 equations was not significantly different from the measured 24-HUNa, while estimated 24-HUNa obtained by the equations derived previously [4,5,7] showed significant differences from measured 24-HUNa (Table 2). 

**Table 2:** Calculated 24-hour urine sodium excretion obtained by six equations.

Equations	Mean ± SD	Mean Difference	*p* *
Kawasaki’s equation (mmol/24-hour)	195.5 ± 59.4	−30.2 ± 54.6	<0.001
Tanaka’s equation (mmol/24-hour)	153.1 ± 40.0	12.2 ± 53.8	0.001
INTERSALT equation (mmol/24-hour)	131.7 ± 31.6	33.6 ± 53.6	<0.001
Linear equation (mmol/24-hour)	159.6 ± 56.5	5.7 ± 53.7	0.113
Quadratic equation (mmol/24-hour)	160.1 ± 58.4	5.2 ± 54.6	0.154
Cubic equation (mmol/24-hour)	164.4 ± 76.6	0.9 ± 67.1	0.836

Mean difference was calculated by subtracting estimated from measured 24-hour urine sodium excretion; * Estimated 24-hour urine sodium excretion by each equation was compared to measured 24-hour urine sodium excretion (mean ± SD, 165.3 ± 65.5 mmol/24-hour) by paired *t*-test.

**Figure 4:**
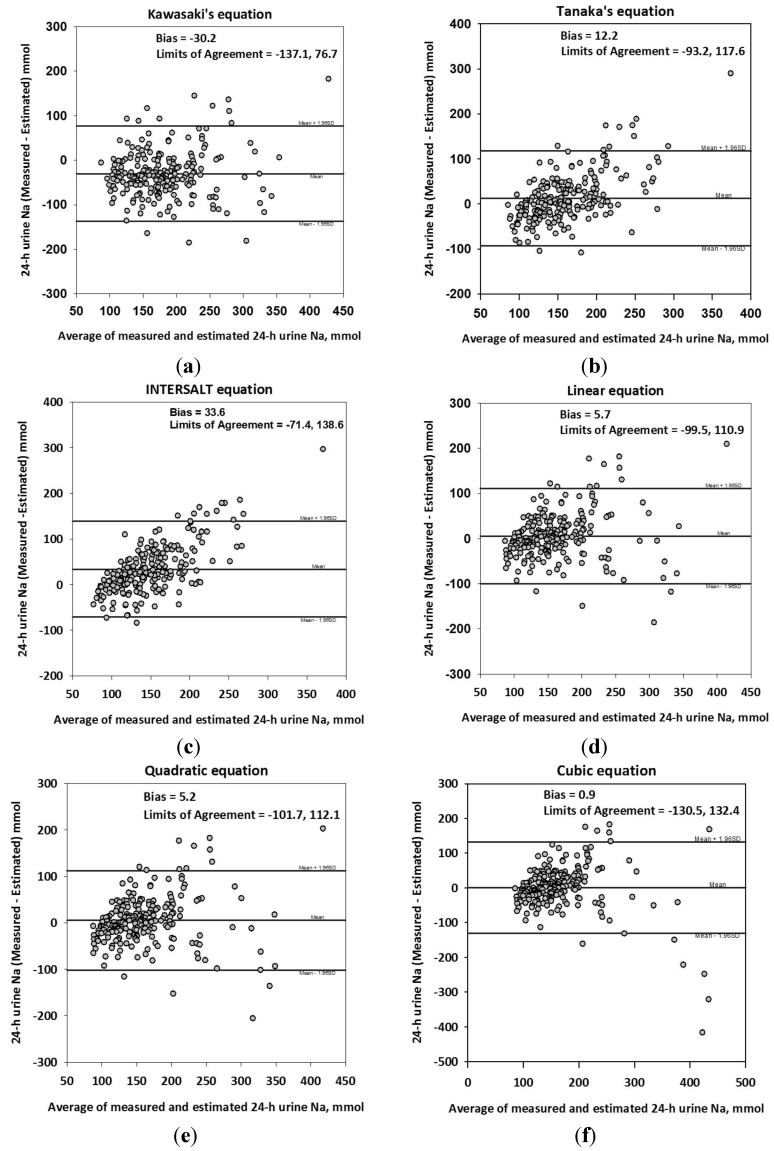
Bland-Altman analysis for agreement of the six equations. (**a**) Kawasaki’s equation; (**b**) Tanaka’s equation; (**c**) INTERSALT equation; (**d**) linear equation; (**e**) quadratic equation; (**f**) cubic equation.

In the Bland-Altman analysis of the difference between measured and estimated 24-HUNa obtained by the six equations, the standard deviations were 54.6 for the Kawasaki *et al*.’s equation, 53.8 for the Tanaka *et al*.’s equation, 53.6 for the INTERSALT equation, 53.7 for the linear equation, 54.6 for the quadratic equation, and 67.1 for the cubic equation, showing large limits of agreement between the methods (Figure 4). There was significant tendency of over- and under-estimation depending on the average of measured and estimated 24-HUNa except for the equation of Kawasaki *et al*. The correlation between difference and average was 0.123 (95% CI −0.008 to 0.262) for the equation of Kawasaki *et al*., 0.592 (95% CI 0.414 to 0.596) for the equation of Tanaka *et al*., 0.699 (95% CI 0.496 to 0.651) for the INTERSALT equation, 0.185 (95% CI 0.057 to 0.323) for the linear equation, 0.145 (95% CI 0.014 to 0.281) for the quadratic equation, and −0.188 (95% CI −0.298 to −0.054) for the cubic equation. In addition, the percent error was 64.7% for Kawasaki *et al*.’s equation, 63.8% for Tanaka *et al*.’s equation, 63.5% for the INTERSALT equation, 63.7% for the linear equation, 64.7% for the quadratic equation, and 79.5% for the cubic equation.

**Figure 5:**
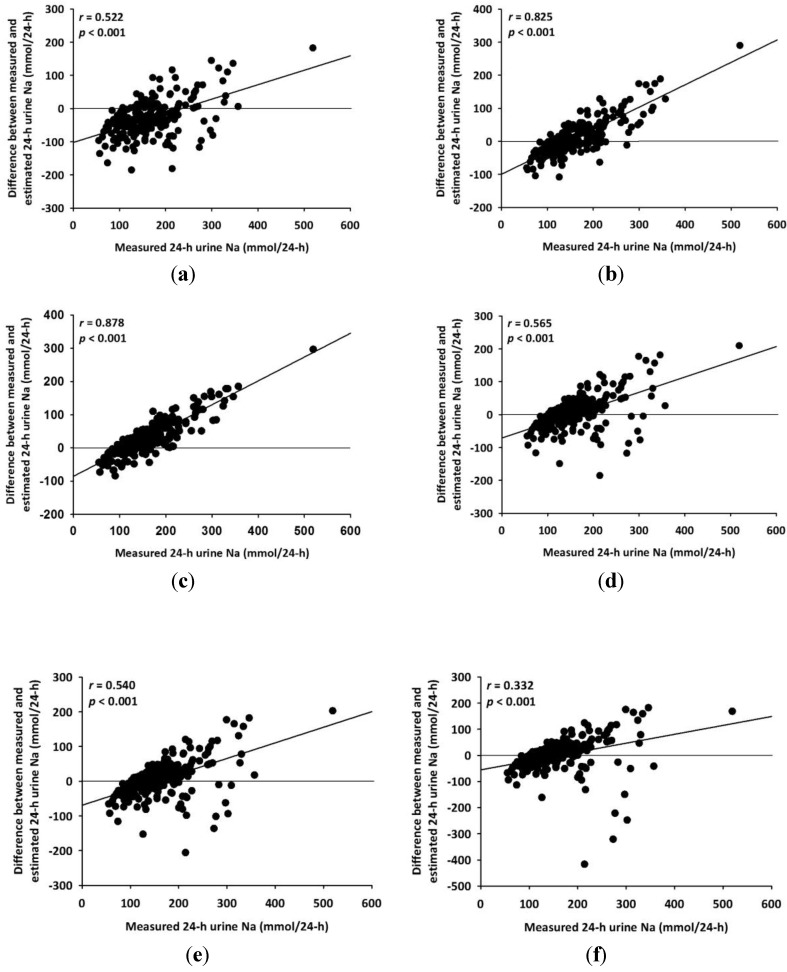
Relationship between the differences of measured and estimated 24-hour urine sodium against measured 24-hour urine sodium. (**a**) Kawasaki’s equation; (**b**) Tanaka’s equation; (**c**) INTERSALT equation; (**d**) linear equation; (**e**) quadratic equation; and (**f**) cubic equation.

The difference between measured and estimated 24-HUNa significantly increased with increases in measured 24-HUNa, while it significantly decreased with decreases in measured 24-HUNa (Figure 5). 

When the subjects were divided into high and low 24-HUNa groups (above and below the median value of 156 mmol/24 h), 24-HUNa values were under-estimated compared to measured 24-HUNa except for Kawasaki’s equation in the high 24-HUNa group. In the low 24-HUNa group, the equations except for the INTERSALT equation over-estimated 24-HUNa (Table 3).

**Table 3:** Difference of estimated and measured 24-hour urine sodium excretion by levels of measured 24-hour urine sodium excretion.

Equations	24-hour urine sodium <156 mmol/24-hour			24-hour urine sodium ≥ 156 mmol/24-hour		
	Mean = 116.6 ± 25.4 mmol/24-hour			Mean = 213.1 ± 57.0 mmol/24-hour		
	Mean ± SD	Mean difference	*p* *	Mean ± SD	Mean difference	*p* *
Kawasaki’s equation (mmol/24-hour)	166.8 ± 39.4	−50.2 ± 38.9	<0.001	223.7 ± 62.3	−10.6 ± 60.5	0.065
Tanaka’s equation (mmol/24-hour)	136.3 ± 25.9	−19.7 ± 29.9	<0.001	169.5 ± 38.9	43.6 ± 53.6	<0.001
INTERSALT equation (mmol/24-hour)	116.5 ± 21.7	0.1 ± 28.8	0.974	146.6 ± 32.7	66.5 ± 51.9	<0.001
Linear equation (mmol/24-hour)	133.6 ± 30.0	−17.0 ± 32.4	<0.001	185.1 ± 64.4	28.0 ± 60.8	<0.001
Quadratic equation (mmol/24-hour)	133.7 ± 29.7	−17.1 ± 32.2	<0.001	186.0 ± 67.5	27.1 ± 62.7	<0.001
Cubic equation (mmol/24-hour)	134.2 ± 29.9	−17.5 ± 32.4	<0.001	194.0 ± 95.0	19.1 ± 85.2	0.019

Subjects were divided into high and low measured 24-hour urine sodium levels. * *p* value by paired *t*-test.

## 4. Discussion

The most reliable method of estimating sodium intake is the 24-hour urine collection which is used in many clinical and epidemiologic studies [3]. However, surveys of sodium intake by 24-hour urine collection have been performed in relatively small samples <1000 because of the difficulties associated with urine collection [9,15]. Thus, a more convenient method of sodium intake assessment such as the SU collection method has been sought [15]. 

As with previous studies which developed 24-HUNa estimation equations with SU samples, the equations developed in the present study yielded 24-HUNa values with fairly high correlations to the measured 24-HUNa in the validation study. Furthermore, there were no significant differences between measured and estimated 24-HUNa by our equations in the validation study. The differences between measured and estimated 24-HUNa by our equations were <6 mmol/24-hour. The 24-HUNa estimated by the previously derived equations [4,5,7] had fairly high correlations with measured 24-HUNa as well, while there was a significant difference between estimated and measured 24-HUNa. A significant bias of the INTERSALT equation is quite different from results of a previous study [16]. Kawasaki’s and Tanaka’s equations were developed from Asian populations. On the contrary, the INTERSALT equation was developed from a Western population. Thus, there is a possibility of ethnic difference, requiring future studies. Another explanation is an age difference of studied populations. Although there were high correlations, we found an important limitation of equations using SU sample in the estimation of 24-HUNa.

Many previous studies suggested SU collection method in the estimation of 24-HUNa as a substitute of 24-hour urine collection method, because there was high correlation and small bias between estimated and measured 24-HUNa [4,5,7,17,18]. However, when the feasibility of SU collection method as a substitute of 24-hour urine collection method is evaluated, tests of agreement between methods should be performed. Correlation analysis only is not enough. In this regard, the Bland-Altman method is commonly used to determine agreement between methods [19]. Using the Bland-Altman method, Brown *et al*. suggested that the SU collection method is a useful alternative to 24-hour urine collection because there was small bias between two methods (−1.6 mmol for men and 2.3 mmol for women) [7]. In the present study, the cubic equation developed by us showed relatively small bias (0.9 mmol). When we consider the previous and our result, the SU collection method seems to be useful substitute of 24-hour urine collection method. However, limits of agreement and percentage errors should be considered in the Bland-Altman method [20,21]. In the study of Brown *et al*. the limits of agreement was similar to ours, more than 100 mmol [7]. Although percentage errors were not presented in the study of Brown *et al*. they were more than 60% in the present study. The limits of agreement and percentage errors are too big to use SU collection method as a substitute of 24-hour urine collection method in the estimation of individual’s sodium intake.

Furthermore, under- or over-estimation of 24-HUNa according to the level of measured 24-HUNa is also an important limitation why SU collection methods cannot be used as a substitute of 24-hour urine collection methods in the assessment of an individual’s sodium intake. In the study of Tanaka *et al*. there was a significant difference between estimated and measured 24-HUNa (24 mmol/day, *p <* 0.001), when their equation was applied to an external population [5]. From that finding, they also noted that the SU collection method is not suitable for estimating an individual’s 24-HUNa values. 

The aforementioned results mean that the assessment of sodium intake by the SU collection method may lead to incorrect conclusions regarding the effect of sodium intake on disease occurrence, progression, and outcomes. A recent study [6] used the SU collection method in the evaluation of the relationship between sodium intake and cardiovascular events. The authors used morning fasting urine samples (not exactly second morning urine) and Kawasaki *et al*.’s equation in the estimation of 24-HUNa [6]. Although the correlation between measured and estimated 24-HUNa by Kawasaki *et al*.’s equation was also significant in the PURE (Prospective Urban Rural Epidemiological) population (*r =* 0.55) [6], the bias of equations using the SU collection method in the estimation of 24-HUNa, which was evident in the present study, may lead to the wrong conclusion. Thus, the conclusion of a J-shaped association between estimated urinary sodium excretion and cardiovascular events does not mean that low sodium intake is associated with high cardiovascular events. Another example is a study that evaluated an association between central hemodynamics and sodium intake [22]. In that study, sodium intake was estimated from SU samples by the equation of Tanaka *et al*. However, as the latter equation appeared inadequate to substitute 24-hour urine collection method in the assessment of individual’s sodium intake in the validation study of Tanaka *et al*. and the present study, their conclusion should be interpreted with a caution.

The present study has limitations. The previously developed equations [4,5,7] were developed from second morning and randomly collected urine specimens, respectively. We used first-morning voided urine specimens in the estimation of 24-HUNa by the aforementioned equations. Although the correlation between spot and 24-hour urine on the excretion of sodium and creatinine was significant, there was circadian variation in the urinary sodium and creatinine excretion, and sodium/creatinine ratio [23,24]. The difference in the collection times may be a cause of differences observed between estimated and measured 24-HUNa in the validation test. The circadian variations in the amount of urinary sodium excretion depends on the amount of sodium intake [25]. The urinary sodium and creatinine excretion depend on the meals [26]. Thus, Mann *et al*. recommended collection of SU samples at the midpoint of 24-hour urine collection for the highest correlation between measured and estimated 24-HUNa [17]. Although Mann *et al*. showed the highest correlation of estimated 24-HUNa obtained from later afternoon and early evening SU, which was collected prior to the evening meal and near to the midpoint of 24-hour urine collection, to measured 24-HUNa, they did not present the result of method comparison analysis [17]. In the present study, the evening SU collected around the midpoint of 24-hour urine collection had lower correlation coefficient of SUNa/SUCr compared to first-morning SU. However, we could not avoid the effect of the evening meal on the urinary creatinine and sodium excretion because evening SU was collected after the evening meal. In addition, hypertensive subjects have higher nighttime urinary sodium excretion than normotensive subjects [27]. In the subgroup analysis of normotensive and hypertensive subjects, the significant tendency of under- and over-estimation was persistent. The second limitation is the collection of single SU in the estimation of 24-HUNa. In the present study, when we combined first-morning and evening SU, the correlation coefficient of XNa was 0.731 (data not shown), which was higher than that of single SU. We could not test the validation of the equation from the combined first-morning and evening SU because we did not have evening SU samples to test in the validation group. With the hypothesis that using multiple SU samples is better than using a single SU, we are investigating the usefulness of multiple SU (first-morning, afternoon, and evening SU, or 5 days morning first SU) in the estimation of 24-HUNa.

## 5. Conclusions

Estimation of 24-HUNa from single SU by equations tested in the present study was inadequate for the estimation of an individual’s sodium intake. However, future studies for various SU collection methods in the estimation of 24-HUNa, such as collection of multiple SU samples, and the usefulness of SU collection methods in the estimation of population sodium intake, are warranted.

## Supplementary Files

Supplementary File 1 Supplementary Information (DOCX, 2085 KB)
