# Supplementary material for: Estimation of 24-Hour Urinary Sodium Excretion Using Spot Urine Samples
Source: Nutrients. 2014 Jun 20;6(6):2360–75. doi: 10.3390/nu6062360 (PMC4073156; doi:10.3390/nu6062360)

**Supplementary Information**

**Table S1.** Demographic and clinical characteristics of Group 2 subjects.

| **Variables** | **Normotensives**  ***N* = 117** | **Hypertensives**  ***N* = 107** |
| --- | --- | --- |
| Age (years) | 49.0 ± 11.1 | 53.1 ± 10.2 |
| Men (%) | 28 (23.9) | 61 (57.0) |
| Diabetes (%) | 3 (2.6) | 8 (7.5) |
| Body weight (kg) | 59.4 ± 9.1 | 67.9 ± 12.1 |
| Height (cm) | 161.2 ± 7.0 | 164.4 ± 7.9 |
| Serum sodium (mmol/dL) | 140.3 ± 2.2 | 140.5 ± 2.6 |
| Serum potassium (mmol/dL) | 4.4 ± 0.5 | 4.3 ± 0.4 |
| Serum creatinine (mg/dL) | 0.76 ± 0.15 | 0.84 ± 0.17 |
| 24-hour urine sodium (mmol/24-hour) | 147.9 ± 51.1 | 184.3 ± 73.9 |

Data are expressed as mean ± standard deviation, or numbers and percentages in parentheses, as appropriate.

**Table S2.** Calculated 24-hour urine sodium excretion obtained by six equations in normotensive subjects.

| **Equations** | **Mean ± SD** | **Mean difference** | ***p* *** |
| --- | --- | --- | --- |
| Kawasaki’s equation (mmol/24-hour) | 181.5 ± 45.3 | -33.9 ± 39.7 | <0.001 |
| Tanaka’s equation (mmol/24-hour) | 145.9 ± 29.8 | 2.0 ± 39.9 | 0.594 |
| INTERSALT equation (mmol/24-hour) | 120.7 ± 23.7 | 27.1 ± 44.4 | <0.001 |
| Linear equation (mmol/24-hour) | 145.7 ± 36.9 | 2.2 ± 37.7 | 0.523 |
| Quadratic equation (mmol/24-hour) | 145.6 ± 36.7 | 2.3 ± 37.7 | 0.515 |
| Cubic equation (mmol/24-hour) | 145.8 ± 36.6 | 2.1 ± 37.7 | 0.552 |

Mean difference was calculated by subtracting estimated from measured 24-hour urine sodium excretion.
* Estimated 24-hour urine sodium excretion by each equation was compared to measured 24-hour urine sodium excretion (mean ± SD, 147.9 ± 51.1 mmol/24-hour) by paired *t*-test.

**Table S3.** Calculated 24-hour urine sodium excretion obtained by six equations in hypertensive subjects.

| **Equations** | **Mean ± SD** | **Mean difference** | ***p* *** |
| --- | --- | --- | --- |
| Kawasaki’s equation (mmol/24-hour) | 210.5 ± 68.9 | −26.2 ± 67.1 | <0.001 |
| Tanaka’s equation (mmol/24-hour) | 160.9 ± 42.2 | 23.5 ± 64.0 | <0.001 |
| INTERSALT equation (mmol/24-hour) | 143.7 ± 34.8 | 40.6 ± 61.5 | <0.001 |
| Linear equation (mmol/24-hour) | 174.8 ± 69.1 | 9.5 ± 66.9 | 0.145 |
| Quadratic equation (mmol/24-hour) | 175.9 ± 72.2 | 8.4 ± 68.5 | 0.205 |
| Cubic equation (mmol/24-hour) | 184.7 ± 100.4 | −0.3 ± 88.9 | 0.969 |

Mean difference was calculated by subtracting estimated from measured 24-hour urine sodium excretion.
* Estimated 24-hour urine sodium excretion by each equation was compared to measured 24-hour urine sodium excretion (mean ± SD, 184.3 ± 73.9 mmol/24-hour) by paired *t*-test.

**Figure S1.** Relationship between measured and estimated 24-hour urine sodium obtained by six equations in normotensive subjects. (**a**) Kawasaki’s equation, (**b**) Tanaka’s equation, (**c**) INTERSALT equation, (**d**) linear equation, (**e**) quadratic equation, and (**f**) cubic equation.

a)
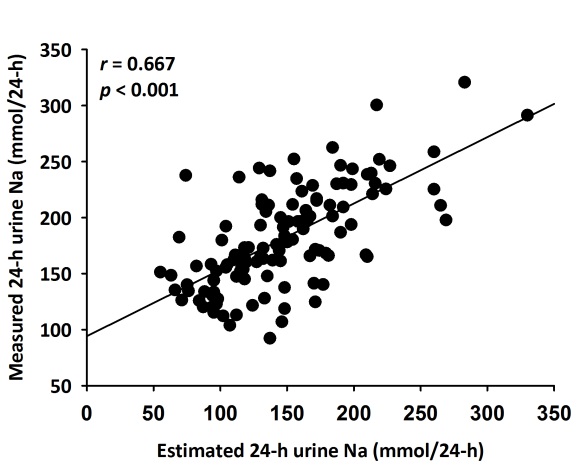
 b)
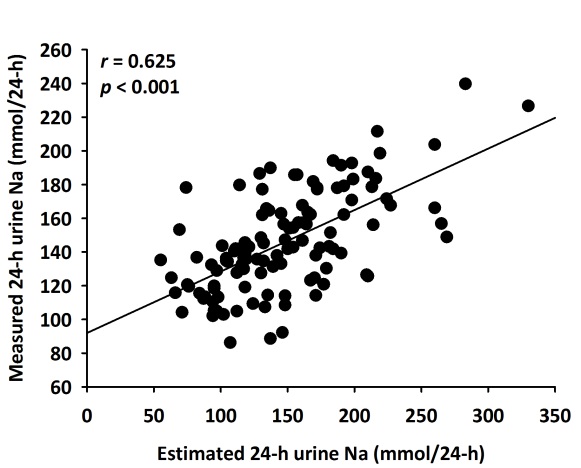


c)
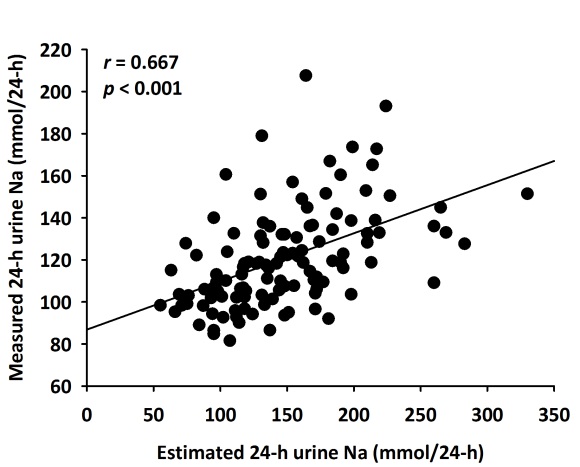
 d)
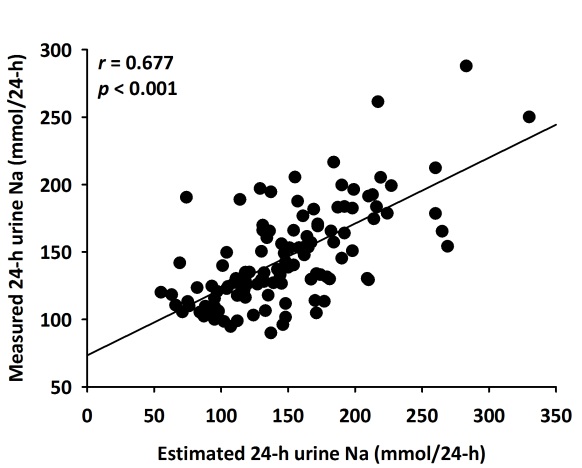


e)
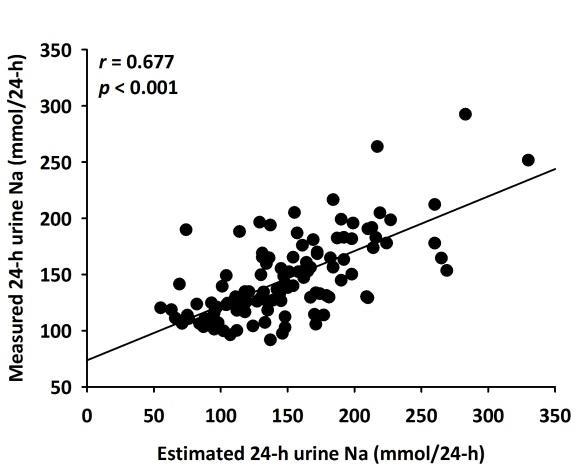
 f)
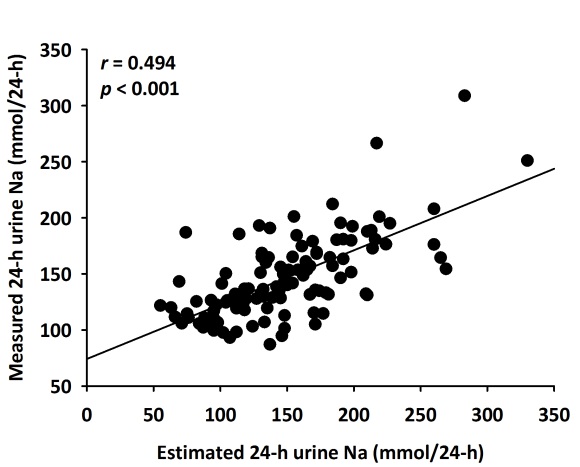


**Figure S2.** Bland-Altman analysis for agreement of the six equations in normotensive subjects. (**a**) Kawasaki’s equation, (**b**) Tanaka’s equation, (**c**) INTERSALT equation, (**d**) linear equation, (**e**) quadratic equation, (**f**) cubic equation.

a)
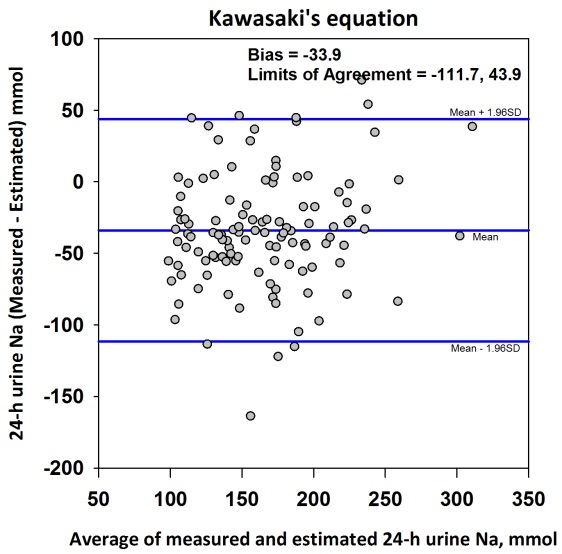
 b)
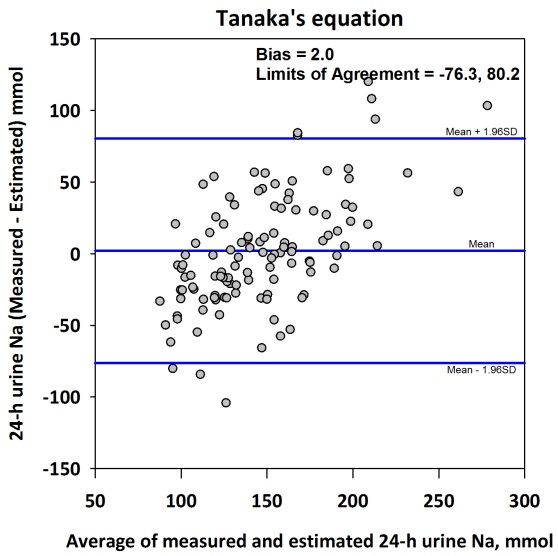


c)
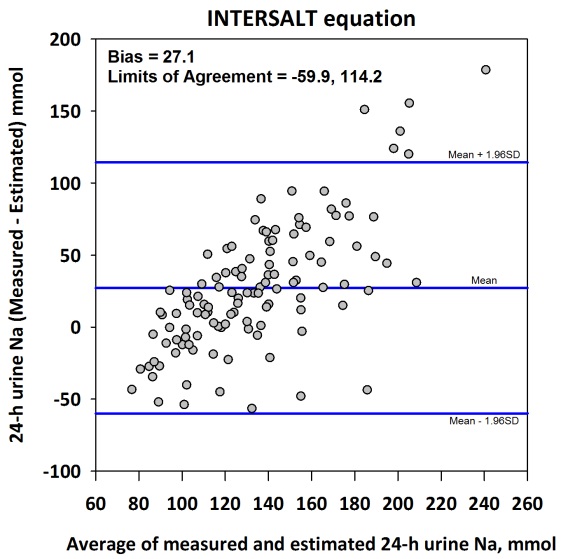
 d)
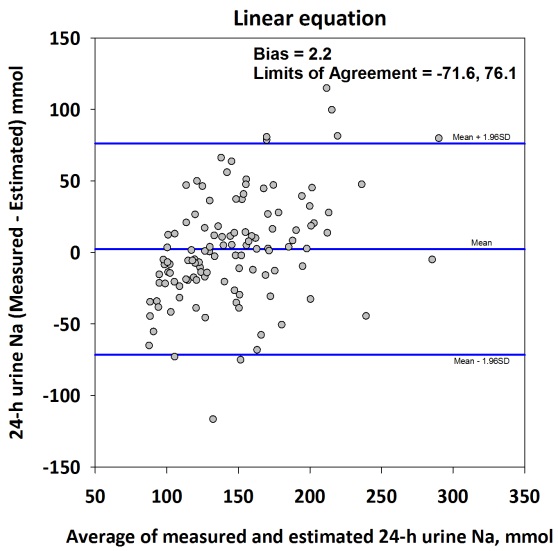


e)
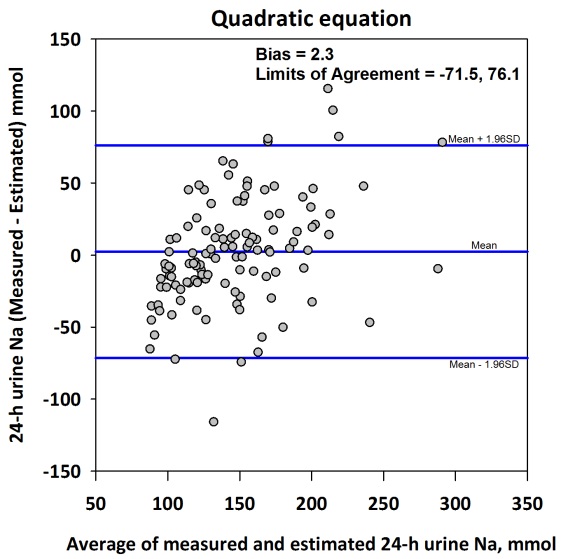
 f)
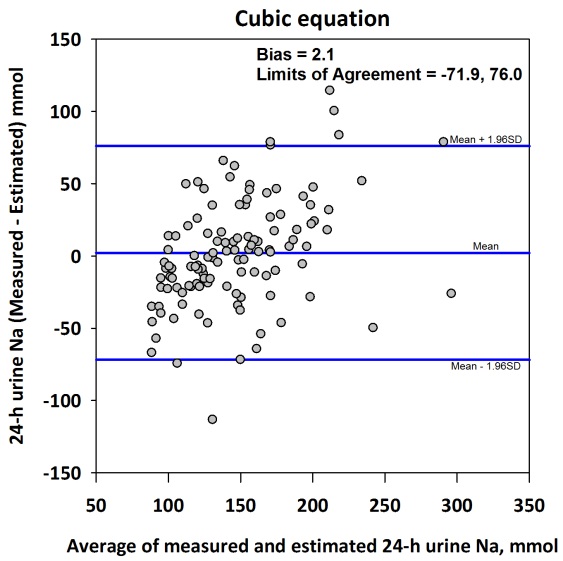


**Figure S3.** Relationship between the differences of measured and estimated 24-hour urine sodium against measured 24-hour urine sodium in normotensive subjects. (**a**) Kawasaki’s equation, (**b**) Tanaka’s equation, (**c**) INTERSALT equation, (**d**) linear equation, (**e**) quadratic equation, and (**f**) cubic equation.

a)
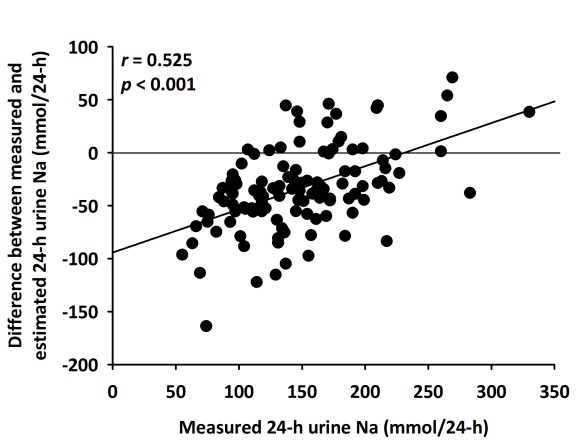
 b)
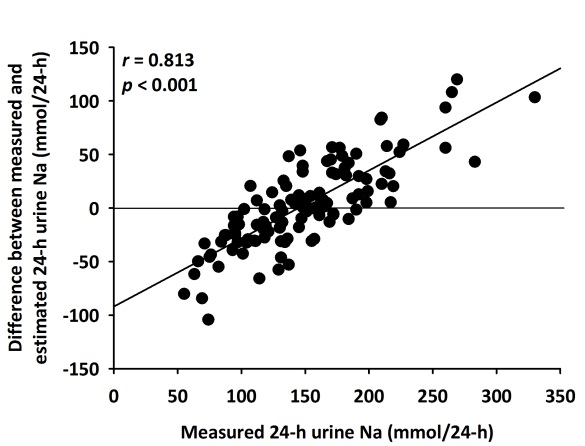


c)
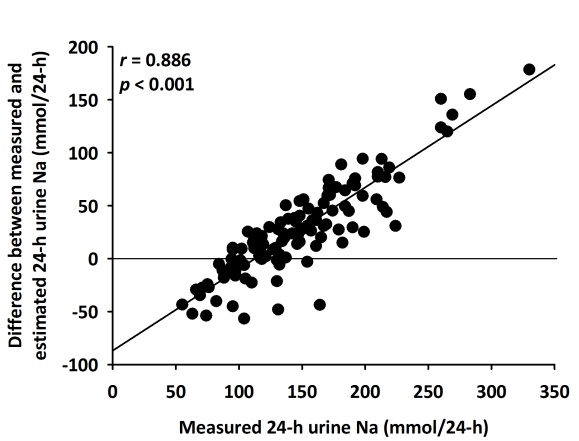
 d)
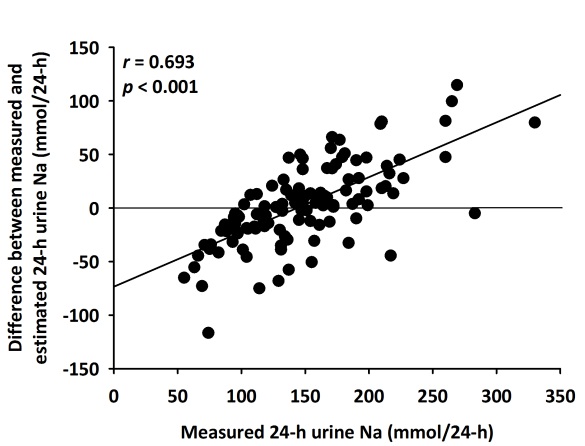


e)
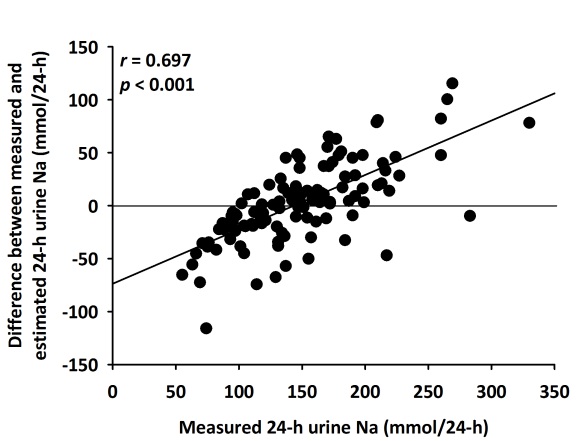
 f)
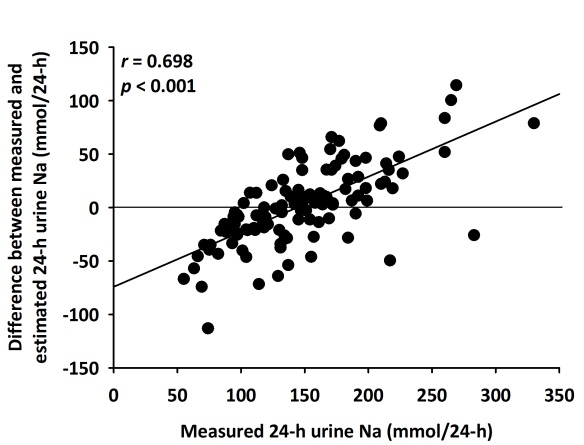


**Figure S4.** Relationship between measured and estimated 24-hour urine sodium obtained by six equations in hypertensive subjects. (**a**) Kawasaki’s equation, (**b**) Tanaka’s equation, (**c**) INTERSALT equation, (**d**) linear equation, (**e**) quadratic equation, and (**f**) cubic equation

a)
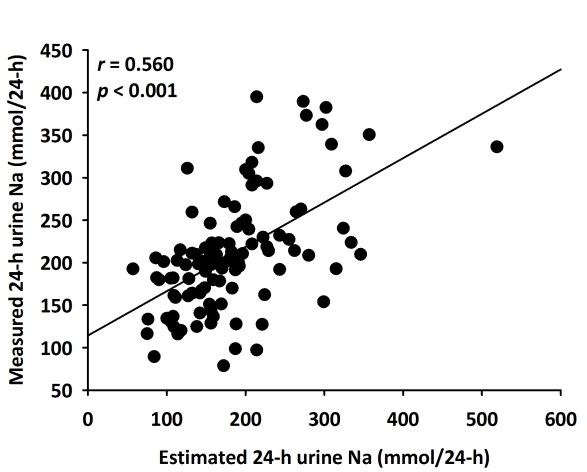
 b)
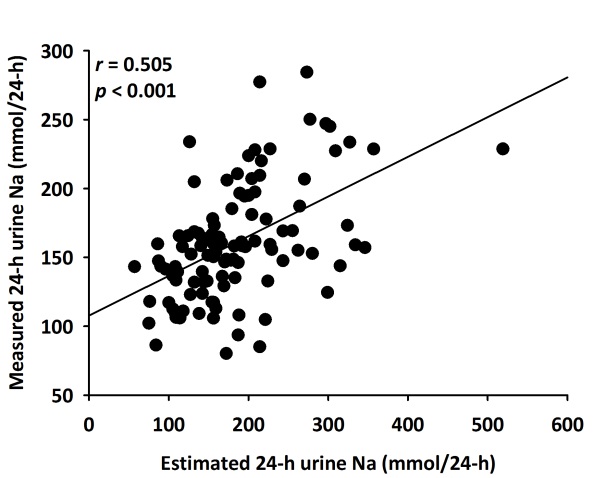


c)
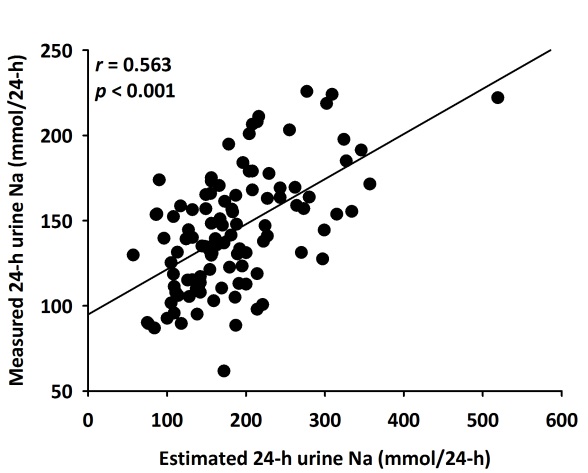
 d)
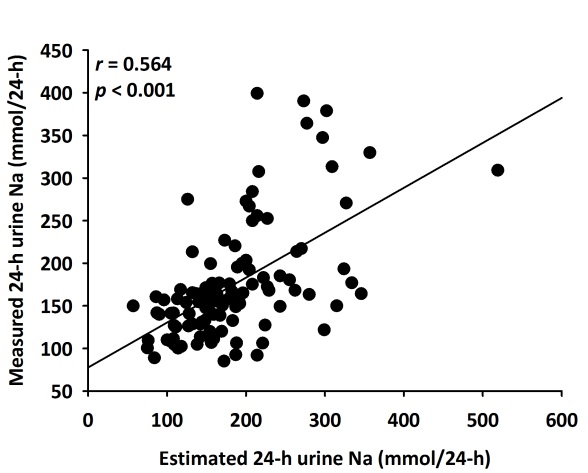


e)
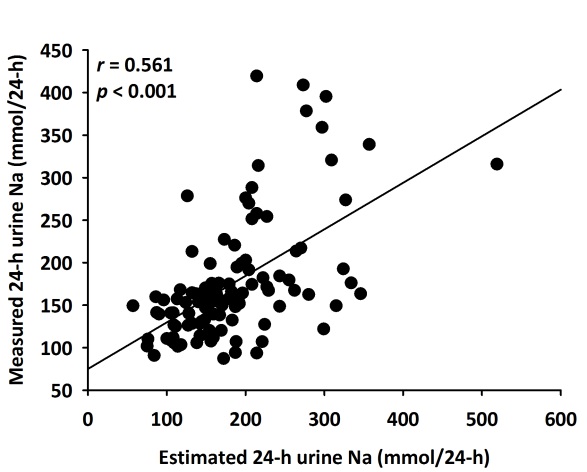
 f)
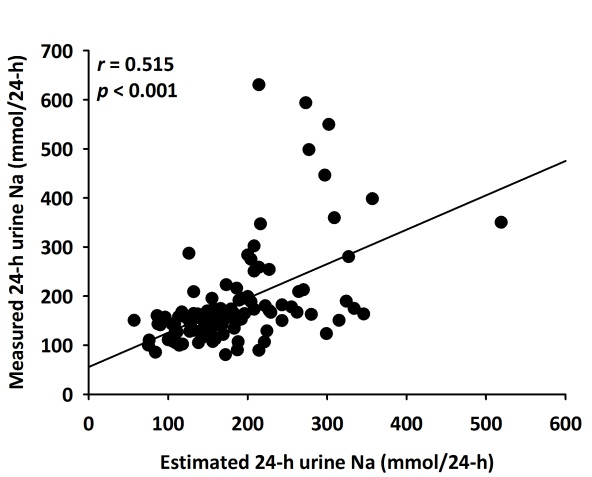


**Figure S5.** Bland-Altman analysis for agreement of the five equations in hypertensive subjects. (**a**) Kawasaki’s equation, (**b**) Tanaka’s equation, (**c**) INTERSALT equation, (**d**) linear equation, (**e**) quadratic equation, (**f**) cubic equation.

a)
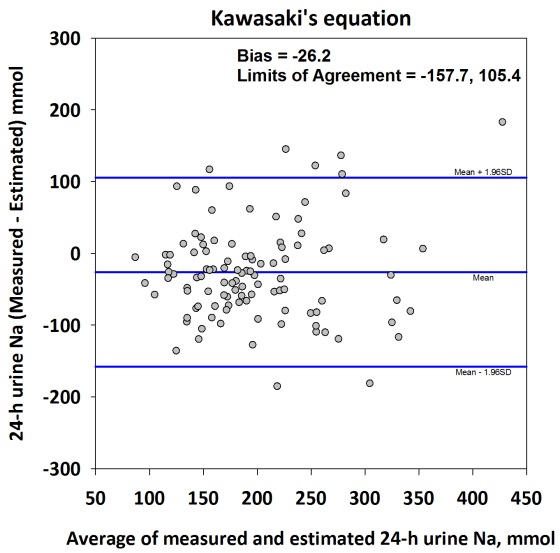
 b)
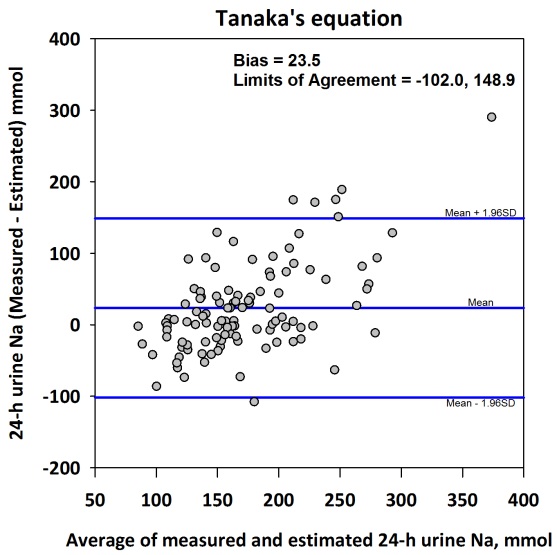


c)
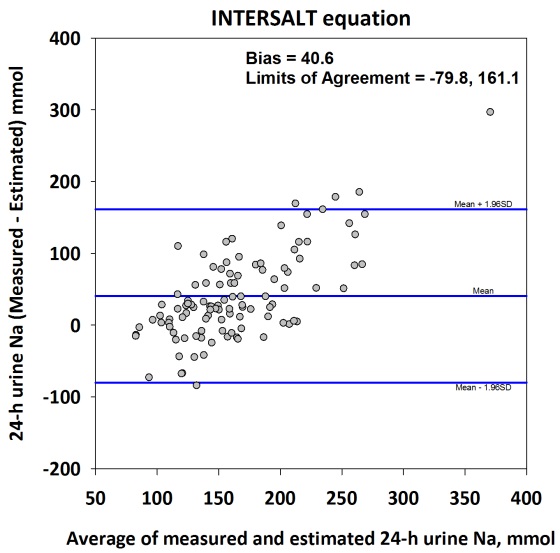
 d)
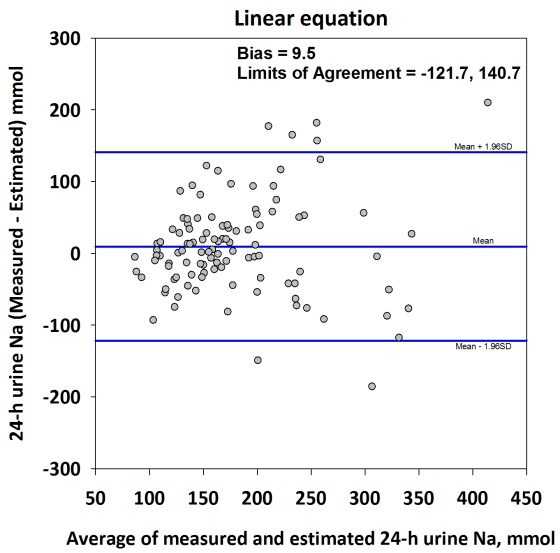


e)
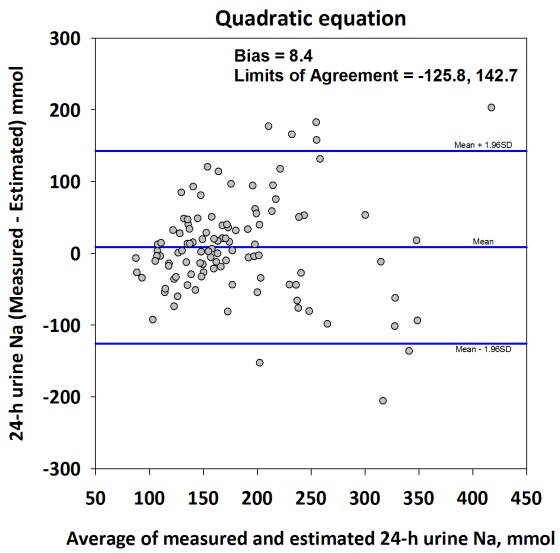
 f)
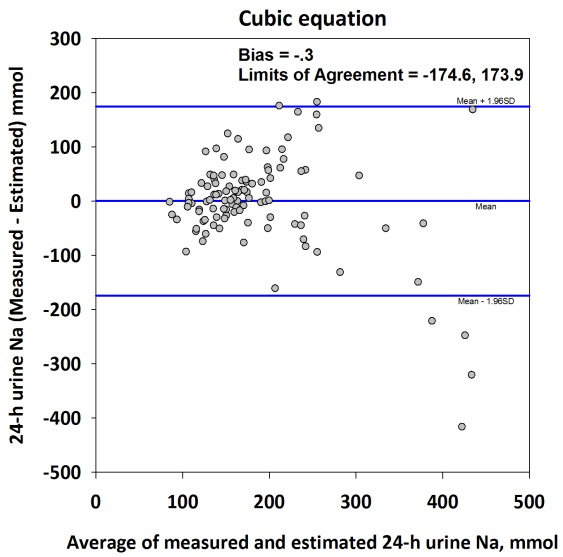


**Figure S6.** Relationship between the differences of measured and estimated 24-hour urine sodium against measured 24-hour urine sodium in hypertensive subjects. (**a**) Kawasaki’s equation, (**b**) Tanaka’s equation, (**c**) INTERSALT equation, (**d**) linear equation, (**e**) quadratic equation, and (**f**) cubic equation.

a)
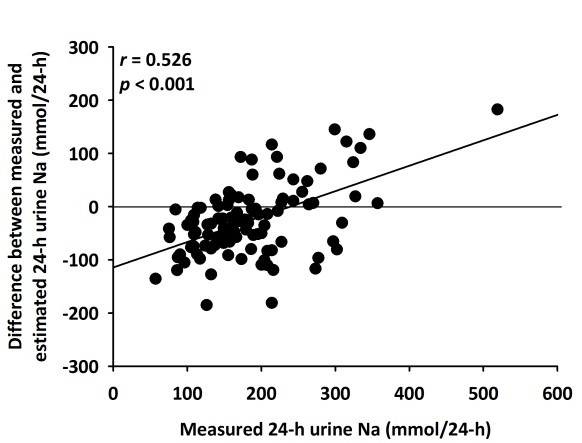
 b)
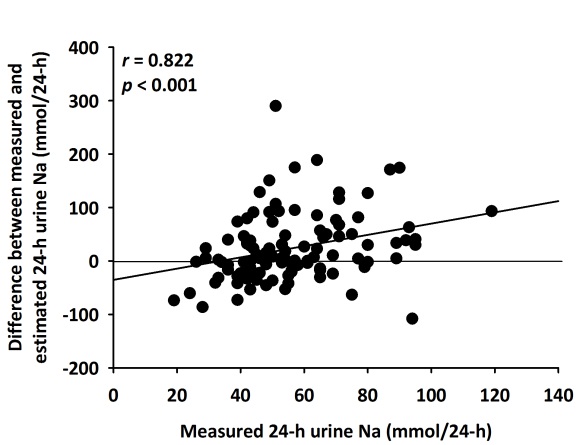


c)
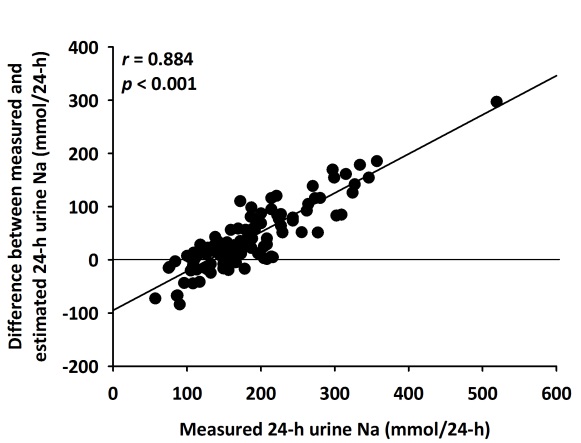
 d)
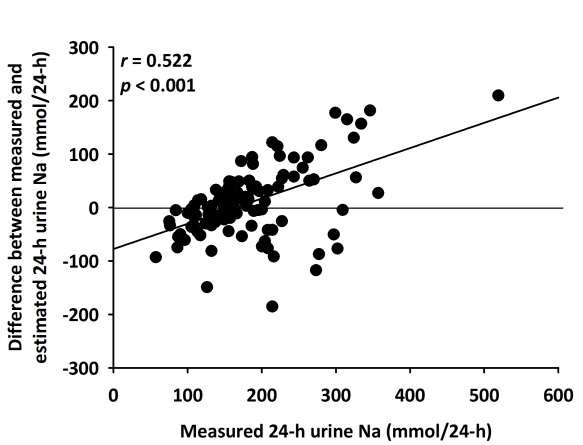


e)
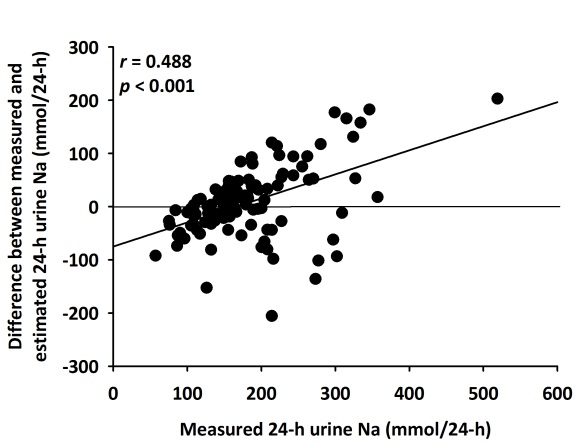
 f)
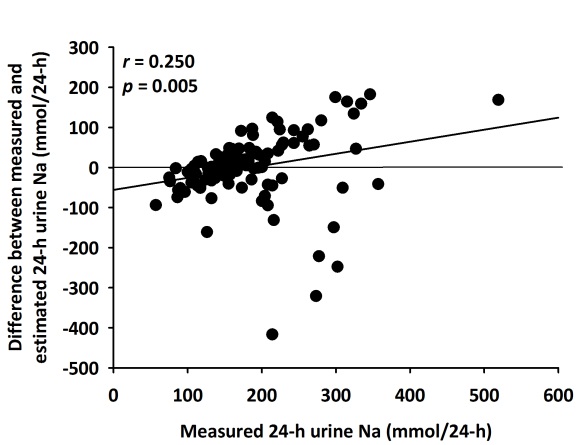

Supplement: Supplementary File 1 — Supplementary Information (DOCX, 2085 KB) [file nutrients-06-02360-s001.docx]
